# Development and Testing of a Mobile App for Management of Gestational Diabetes in Nepal: Protocol for a User-Centered Design Study and Exploratory Randomized Controlled Trial

**DOI:** 10.2196/59423

**Published:** 2024-10-21

**Authors:** Lauren T Berube, Archana Shrestha, Abha Shrestha, Jean-Francois Daneault, Prabin Raj Shakya, Meghnath Dhimal, Roman Shrestha, Shristi Rawal

**Affiliations:** 1 Public Health Nutrition Program Department of Epidemiology New York University School of Global Public Health New York, NY United States; 2 Institute for Implementation Science and Health Kathmandu Nepal; 3 Department of Public Health Kathmandu University School of Medical Sciences Dhulikhel Nepal; 4 Department of Chronic Disease Epidemiology Center of Methods for Implementation and Prevention Science Yale School of Public Health New Haven, CT United States; 5 Department of Obstetrics and Gynecology Dhulikhel Hospital-Kathmandu University Hospital Dhulikhel Nepal; 6 Department of Rehabilitation and Movement Sciences School of Health Professions Rutgers University Newark, NJ United States; 7 Lab of Computer Science Massachusetts General Hospital Boston, MA United States; 8 Nepal Health Research Council Kathmandu Nepal; 9 Department of Allied Health Sciences University of Connecticut Storrs, CT United States; 10 Department of Clinical and Preventive Nutrition Sciences School of Health Professions Rutgers University Newark, NJ United States

**Keywords:** gestational diabetes mellitus, mobile health, mHealth, self-management, pregnancy, maternal and child health, South Asia, Nepal, low- and middle-income country, mobile phone

## Abstract

**Background:**

The prevalence of gestational diabetes mellitus (GDM) is increasing, particularly in low- and middle-income countries (LMICs) like Nepal. GDM self-management, including intensive dietary and lifestyle modifications and blood glucose monitoring, is critical to maintain glycemic control and prevent adverse outcomes. However, in resource-limited settings, several barriers hinder optimal self-management. Mobile health (mHealth) technology holds promise as a strategy to augment GDM treatment by promoting healthy behaviors and supporting self-management, but this approach has not yet been tested in any LMIC.

**Objective:**

This report describes the protocol to develop a culturally tailored mHealth app that supports self-management and treatment of GDM (GDM–Dhulikhel Hospital [GDM-DH] app, phase 1) and test its usability and preliminary efficacy (phase 2) among patients with GDM in a periurban hospital setting in Nepal.

**Methods:**

The study will be conducted at Dhulikhel Hospital in Dhulikhel, Nepal. In the development phase (phase 1), a prototype of the GDM-DH app will be developed based on expert reviews and a user-centered design approach. To understand facilitators and barriers to GDM self-management and to gather feedback on the prototype, focus groups and in-depth interviews will be conducted with patients with GDM (n=12), health care providers (n=5), and family members (n=3), with plans to recruit further if saturation is not achieved. Feedback will be used to build a minimum viable product, which will undergo user testing with 18 patients with GDM using a think-aloud protocol. The final GDM-DH app will be developed based on user feedback and following an iterative product design and user testing process. In the randomized controlled trial phase (phase 2), newly diagnosed patients with GDM (n=120) will be recruited and randomized to either standard care alone or standard care plus the GDM-DH app from 24-30 weeks gestation until delivery. In this proof-of-concept trial, feasibility outcomes will be app usage, self-monitoring adherence, and app usability and acceptability. Exploratory treatment outcomes will be maternal glycemic control at 6 weeks post partum, birth weight, and rates of labor induction and cesarean delivery. Qualitative data obtained from phase 1 will be analyzed using thematic analysis. In phase 2, independent 2-tailed *t* tests or chi-square analyses will examine differences in outcomes between the 2 treatment conditions.

**Results:**

As of July 2024, we have completed phase 1. Phase 2 is underway. The first participant was enrolled in October 2021, with 99 participants enrolled as of July 2024. We anticipate completing recruitment by December 2024 and disseminating findings by December 2025.

**Conclusions:**

App-based lifestyle interventions for GDM management are not common in LMICs, where GDM prevalence is rapidly increasing. This proof-of-concept trial will provide valuable insights into the potential of leveraging mHealth app–based platforms for GDM self-management in LMICs.

**Trial Registration:**

ClinicalTrials.gov NCT04198857; https://clinicaltrials.gov/study/NCT04198857

**International Registered Report Identifier (IRRID):**

DERR1-10.2196/59423

## Introduction

### Background

Gestational diabetes mellitus (GDM), characterized by hyperglycemia that develops during pregnancy, is one of the most common and increasingly prevalent pregnancy complications [1,2]. The International Diabetes Federation estimated that approximately 14% of pregnancies worldwide were affected by GDM in 2021 [2]. A significant proportion of these cases occur in low- and middle-income countries (LMICs), where over 90% of type 2 diabetes (T2D) cases in adults are also reported [3]. In Nepal, a low-income country in South Asia, the national prevalence of GDM is not documented, but regional estimates are high, ranging from 6.6% to over 20% [4-6].

GDM is associated with increased risks of adverse maternal and fetal outcomes, including preeclampsia, birth injuries, labor complications, cesarean delivery, and large for gestational-age babies [7-9]. Although GDM usually resolves after delivery, women with GDM are more likely to develop T2D in the future compared to women with normoglycemic pregnancies [10,11], and fetal exposure to maternal hyperglycemia may predispose offspring to obesity and T2D [12,13]. GDM is also a significant economic burden, with estimated costs from GDM and its downstream consequences ranging from £147 (US $196) million to US $1.6 billion in high-income countries [14,15].

Adequate management of GDM, including diet and lifestyle modifications and frequent self-monitoring of blood glucose, reduces maternal and neonatal complications [16,17]. However, in resource-limited settings like Nepal, GDM management constitutes a significant burden on both patients and the health care system [18,19]. Scalable and cost-effective strategies are thus needed to manage the growing burden of GDM and its consequences in low-resource countries such as Nepal. Barriers to the management of GDM in Nepal exist at multiple levels, including those at the individual (eg, health literacy, lack of knowledge or self-efficacy, and time constraints), interpersonal (eg, cultural practices and family influence on prenatal care decisions) [20-22], and health systems or structural (eg, access to health care, resources, transportation, availability of clinicians, and time for counseling) level [18,23-26]. Mobile health (mHealth) technology provides opportunities to address these multilevel barriers by enhancing self-efficacy and knowledge, promoting self-management behaviors relating to GDM, and facilitating communication between patients and their health care team [27,28]. Evidence from high-income countries shows that mHealth interventions for GDM can improve patient satisfaction and reduce costs [29,30], while achieving better or similar maternal glycemic levels and pregnancy outcomes compared to standard care alone [31]. Nepal has high rates of mobile service penetration among people who are pregnant [32], and prior studies in LMICs support the use of mHealth technology during pregnancy for health promotion, appointment reminders, and improved nutritional status [33-36].

Despite the evidence supporting the use of mHealth technology to improve the self-management of GDM, digital tools for GDM have not been designed to address the unique needs of people who are pregnant and living in LMICs. Innovative and culturally tailored approaches are needed to optimize self-management of GDM in low-resource settings like Nepal [37]. The aim of this paper is to describe the protocol to develop and test the usability, acceptability, and preliminary efficacy of a user-centered, culturally tailored mHealth platform (GDM–Dhulikhel Hospital [GDM-DH] app + web portal) designed to support self-management and treatment of GDM among patients in a periurban hospital setting in Nepal.

### Objective and Hypothesis

In the first phase of the study, our objective is to develop an app prototype for the GDM-DH platform, gather user feedback on the proposed GDM-DH platform, and identify barriers and facilitators to its uptake. In the second phase, we will evaluate the preliminary efficacy of the GDM-DH platform alongside standard care, compared to standard care alone, on perinatal health outcomes, including maternal blood glucose levels, birth weight, and neonatal hypoglycemia. We hypothesize that the GDM-DH platform will show good usability and preliminary efficacy for improving perinatal health outcomes.

## Methods

### Study Overview

**Figure 1:**
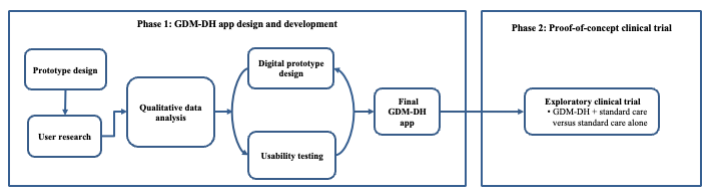
Overview of the study design. GDM-DH: gestational diabetes mellitus-Dhulikhel Hospital.

The study consists of two phases: (1) the design and development of the GDM-DH app platform and (2) a proof-of-concept randomized controlled trial (RCT) to assess its usability, acceptability, and preliminary efficacy (Figure 1). This study will be conducted at Dhulikhel Hospital, a community-based, flagship university hospital of Kathmandu University in Nepal. Dhulikhel Hospital has a catchment population of 1.9 million people and delivers approximately 3000 babies annually [38]. This trial is registered on ClinicalTrials.gov (NCT04198857).

### Ethical Considerations

Institutional approval for this study was granted by Rutgers University Newark Health Sciences institutional review board (Pro2019001883 [phase 1] and Pro2019002841 [phase 2]) and the ethical review board of the Nepal Health Research Council (registration 735/2019). All potential human participants will receive an explanation of the study at screening, including study rationale, procedures, and appointment schedules. Eligible participants will receive a detailed description of the study, including possible risks and benefits, measures to protect privacy, and the right to terminate involvement in the study at any time. Signed written informed consent will be obtained from each participant by a trained research assistant at Dhulikhel Hospital. Enrollment procedures will occur in a private room. To maintain participant confidentiality, documents and forms with identifying information will be stored separately in a locked filing cabinet and in a secure password-protected Box folder (Box Inc), a Health Insurance Portability and Accountability Act (HIPAA)–compliant file storage service. Only select study staff will have access to these files. In phase 1 of the study, participants, including women with GDM and their spouses, will receive a mobile recharge card worth Nepalese rupees 500 (approximately US $3.77) to compensate for their time in the interview, focus group, or usability testing. In phase 2, participants will be compensated with a US $10 prepaid internet data package.

### Phase 1: GDM-DH App Design and Development

The goal of the GDM-DH app is to assist patients in the self-management of GDM by improving their efficacy in adhering to the recommended diet and physical activity regimens and facilitating desired clinical and social support. The app will also help clinicians by providing easily digestible visual displays of patient data and behaviors, which will in turn aid in their clinical decision-making and counseling.

We will take a user-centered approach to design and develop the GDM-DH app that matches the needs and technological sophistication of the target users. As outlined in Figure 1, we will form a multidisciplinary team of experts to design an app prototype, followed by a qualitative or requirement-gathering phase to collect user feedback on the app prototype features and functions. After incorporating and revising the app prototype, we will build a minimum viable product (MVP) and subsequently conduct usability testing. The final GDM-DH app will be developed following an iterative process of product design and user testing. Each step of the app design and development is detailed in the upcoming sections.

### Phase 1A: Prototype Design

As part of the formative phase of the app development, we will first develop a prototype of the GDM-DH app, in collaboration with a multidisciplinary team including experts in GDM, mHealth, and behavior and implementation sciences, as well as health care providers at Dhulikhel Hospital. The content modules and features to be included in the app will be selected based on literature review, relevant theory-based behavioral strategies, and discussion with subject matter experts. The primary educational content will be information on GDM and associated risk factors and health outcomes, clinical and lifestyle management of GDM, dietary and physical activity recommendations for women with GDM, and the benefits of a healthy diet and active lifestyle during pregnancy. Other components will be guided by Bandura’s [39] Social Cognitive Theory (SCT), which postulates that behavior change is guided by several cognitive and social factors, including perceived self-efficacy, social support, outcome expectancies, and perceived barriers and facilitators to the behavior change [40]. Consistent with the SCT framework, we will include features that support self-management of GDM by (1) providing health education; (2) helping patients identify and set target health goals (for diet, physical activity, and glucose levels); (3) enhancing their self-efficacy to meet target goals; and (4) facilitating desired support from family members.

### Key Domains of the GDM-DH App

#### Self-Management of GDM

The app will provide basic educational information about GDM and the importance of managing GDM for optimal maternal and infant health. The app will also include culturally tailored dietary and physical activity recommendations with relevant recipes and example meal plans. The core component of the app will allow participants to record and self-monitor their blood glucose levels, blood pressure, carbohydrate intake, physical activity, and gestational weight gain. To assist with estimating the carbohydrate intake of meals, the app will include typical portion-size images of common carbohydrate food sources in a staple Nepalese diet. Weight, blood pressure, and blood glucose levels can be manually entered by users either biweekly after clinic testing or daily if they have equipment at home.

Based on the user input data, the GDM-DH app will automatically generate tailored feedback and help users monitor their progress toward target health goals. The app will compare carbohydrate intake, physical activity, gestational weight gain, blood glucose, and blood pressure levels to existing evidence-based guidelines [41-45] via a feedback engine and will generate visual display charts. These charts will summarize diet, physical activity, gestational weight gain, blood glucose, and blood pressure patterns, allowing participants to monitor their alignment and progress toward target goals.

The GDM-DH app will enhance self-efficacy to adhere to recommendations and meet target goals with a variety of multimedia modules (eg, video lessons) that provide appropriate strategies and practice opportunities to problem-solve around barriers to health behaviors. These modules will consider specific cultural, social, and physical environmental challenges that patients with GDM face in adopting a healthy diet and lifestyle.

#### Facilitation of Health Exchange Between Patients and Providers

Providers will have access to a web-based administrative portal that syncs with the patient-facing GDM-DH app and allows them to register a new patient, as well as enter, update, or review clinical and other patient-related information (eg, glucose values, blood pressure, weight, diet entries, clinic history, and notes). This portal is intended to streamline the providers’ workflow, as they can quickly look at patient data visualizations to understand patient behaviors and progress and guide their treatment and counseling accordingly.

#### Family Member Support

The GDM-DH app will also facilitate involvement from family members or friends, who strongly influence prenatal care–related decisions and dietary behaviors in Nepal [20-22]. Via a social network “follow” feature, patients will be able to list 1 or more family members or friends as contacts in the app and give that contact permission to view their logged data and progress summary. This feature will be added to the GDM-DH app to garner social support and offer a source of accountability, motivation, and shared experience.

### Phase 1B: Qualitative User Research

The objective of this qualitative phase is to gather user feedback on the GDM-DH app prototype and understand the perceived facilitators and barriers to GDM self-management. We will recruit patients diagnosed with GDM within the past year from Dhulikhel Hospital to participate in a focus group or semistructured in-depth interview [46]. Eligible patients will be pregnant women who (1) receive antenatal care at Dhulikhel Hospital, (2) receive a GDM diagnosis (within the preceding year), (3) own a smartphone, and (4) can understand and read Nepali. Patients with learning difficulties or vision or hearing impairments will be excluded. Patients with a confirmed GDM diagnosis will be recruited into the study with the help of a senior obstetrician-gynecologist (OB-GYN; coinvestigator in the study) and other staff in the OB-GYN Department at Dhulikhel Hospital. We will recruit 12 women with GDM but plan to recruit further if data saturation is not achieved [47-49]. We will also conduct key informant interviews with clinicians from Dhulikhel Hospital (n=5) and family members of patients with GDM (n=3) to collect feedback on the GDM-DH prototype. Eligibility criteria for family members include being a spouse or direct relative of a patient with GDM who was diagnosed with GDM within the past year at Dhulikhel Hospital. All participants will provide written informed consent.

Before the focus group or in-depth interview, current and previously diagnosed patients with GDM will complete a structured questionnaire assessing sociodemographics and pregnancy-related information. The focus group and in-depth interviews will be developed in Nepali with a set of questions and probes to thoroughly understand the perceived social, cultural, and environmental facilitators and barriers to GDM management, including participants’ views, opinions, and knowledge about GDM management, perceived self-efficacy, and strategies to enhance adherence to lifestyle modifications [50]. We will also collect feedback on the GDM-DH app prototype, including (1) the app dashboard, layout, and navigation; (2) the usefulness of app features; (3) the burden of data entry; (4) the usefulness of educational modules covered; (5) clarity of graphs and data visualizations; and (6) additional features and content. The focus group or interview guide will be developed in consultation with the study team with a study investigator (AS) who has prior experience conducting qualitative studies in this population taking the lead. A trained research coordinator will administer the focus group and in-depth interviews at Dhulikhel Hospital.

Given the clinical applications and setting of our study, the qualitative arm of this research takes a pragmatic worldview. In analyzing the qualitative data, our goal will be to generate practical and actionable insights that can directly inform and improve the GDM-DH platform with the ultimate goal of improving patient outcomes. The focus group and in-depth interview will be audio recorded and transcribed before analysis. Qualitative data will be uploaded onto the NVivo 12 software (Lumivero) for data management and analysis. We will follow Braun and Clarke’s [51] 6-phase approach to thematic analysis, including (1) familiarizing with the data, (2) generating initial codes that will be compiled into a codebook, (3) searching for themes, (4) reviewing themes, (5) defining and naming themes, and (6) producing the final report [51]. Our analysis will use both inductive and deductive approaches. First, we will develop deductive themes based on our interview guide. Next, we will read the transcripts to identify text related to the deductive themes and define new inductive themes that emerge from the data. A codebook will be developed by revisiting the text within the identified themes, defining codes, and reorganizing or grouping those not aligning with existing themes into new ones. A second research analyst will use this codebook to independently code the transcripts. After coding is completed, the 2 research assistants will compare the coding schemes and resolve any discrepancies through mutual agreement, with assistance from the study investigator. A third research analyst will calculate the intercoder reliability, achieving a level of 80%. Multiple investigators will review the data and provide insights based on their content knowledge and expertise.

### Phase 1C: Usability Testing

Incorporating and revising the app prototype based on qualitative user research, we will build the MVP, the simplest possible version of the GDM-DH app, which will retain its most important features and functionalities. The MVP of the GDM-DH app will undergo usability testing with patients with GDM via think-aloud protocol [52]. The eligibility criteria to recruit patients with GDM for usability testing will be the same as the criteria used for phase 1B—qualitative user research. We will use a convenience sampling strategy to recruit 18 participants for the usability testing [53]. All patients will be required to provide written informed consent.

Individual one-on-one usability testing sessions will be conducted in a private space and overseen by 2 facilitators; 1 facilitator will lead the session while a designated note-taker will record users’ verbalizations. Usability testing will consist of a 2-step think-aloud protocol in which the participants will be asked to verbalize their thoughts as they navigate and complete various specified tasks (eg, profile setup, diet entry, review weight visualizations, and open video lesson) on the GDM-DH app [54]. Participants will also be asked to rate the difficulty of completing each task on a 5-point scale ranging from “very easy” to “difficult.” At the end of the usability testing session, participants will complete the System Usability Scale (SUS, scored 0-100), a reliable and widely used 10-item 5-point Likert scale questionnaire for global assessment of systems usability [54]. After the usability testing, we will ask open-ended questions to collect feedback on the app, such as how to improve upon the features and functions of the app. The final version of the GDM-DH app will be developed following an iterative process of product design and user testing and will be used in the proof-of-concept RCT in the second phase of the study.

### Phase 2: Proof-of-Concept RCT

The objective of the RCT is to answer the research question—in women with GDM, does augmenting standard care with the GDM-DH platform, compared to standard care alone, show preliminary efficacy in improving clinical perinatal outcomes, including maternal blood glucose levels, birth weight, and neonatal hypoglycemia?

### Participant Eligibility and Recruitment

Patients who are newly diagnosed with GDM will be recruited from the Obstetric Outpatient Department at Dhulikhel Hospital. Patients who are pregnant and receiving antenatal care at the Obstetric Outpatient Department undergo routine screening for GDM at 24 to 28 weeks of gestation and are diagnosed using the Carpenter-Coustan criteria [55]. Eligible patients must (1) receive GDM diagnosis based on Carpenter-Coustan criteria [55], (2) be aged 18 years or older, (3) be ≤30 weeks gestational age, (4) receive antenatal care at Dhulikhel Hospital, (5) own an Android smartphone, (6) have internet connectivity at home, and (7) understand and read Nepali. Patients with learning difficulties or vision or hearing impairments will be excluded.

### Enrollment and Randomization

**Figure 2:**
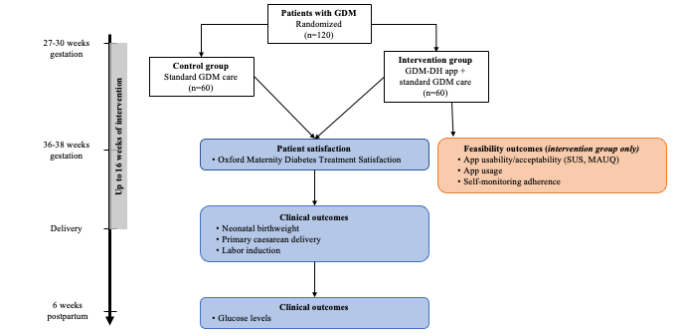
Overview of study design of phase 2 (proof-of-concept clinical trial) and measurement time points. Measures in blue are collected in both study groups, while measures in red are only collected in the intervention group. GDM: gestational diabetes mellitus; GDM-DH: gestational diabetes mellitus-Dhulikhel Hospital; MAUQ: mHealth App Usability Questionnaire; SUS: System Usability Scale.

We will enroll 120 newly diagnosed patients with GDM from the Obstetric Outpatient Department at Dhulikhel Hospital between 24 and 30 weeks gestational age (baseline). At baseline, all participants will be briefed about the study and asked to provide written informed consent to agree to participate in the study. All participants will then complete a structured questionnaire to assess sociodemographics, prepregnancy weight, and lifestyle factors, such as smoking and alcohol use. Next, participants will be randomly assigned in a 1:1 fashion to 1 of the 2 treatment conditions—either GDM-DH app intervention + standard care or standard care only, for up to 16 weeks, starting from baseline until delivery (Figure 2). Random permuted blocks of sizes 4 or 6 will be used using a statistical software in order to prevent treatment imbalance and ensure that participants are allocated to each group equally. The allocation sequence will be concealed from the research assistant and research nurse using sequentially numbered opaque sealed envelopes. Due to the nature of the study, clinicians and participants will not be blinded to the group allocation.

### Standard Care

Standard care for patients with GDM at Dhulikhel Hospital entails a counseling session with an OB-GYN physician, which covers basic disease information, dietary and physical activity recommendations for GDM management, target blood glucose levels before and after meals, and the course of treatment. The primary treatment for GDM includes modifications to diet and physical activity. Participants consult a dietitian and a physical therapist to develop a diet and physical activity plan based on prepregnancy weight and GDM disease severity. In addition to setting carbohydrate goals, major dietary recommendations include eating small meals throughout the day, lowering sugar and refined carbohydrate intake, and increasing the intake of vegetables and whole grains. Patients are provided with leaflets and brochures in addition to verbal information about managing GDM with diet and physical activity. As per the standard care protocol, patients with GDM are asked to visit the Obstetric Outpatient Department for glucose testing every 2 weeks, and after each testing, blood glucose levels are recorded in paper booklets assigned to each patient. If feasible, patients are also encouraged to buy a glucometer and perform daily blood glucose testing at home. The OB-GYN physicians monitor the blood glucose levels across testing and, if needed, may prescribe oral hypoglycemic medications or insulin to the patient over the course of treatment.

### Study Intervention

In addition to standard care, participants in the intervention group will use the GDM-DH app to support self-management behaviors. At baseline, participants in the intervention group will have the GDM-DH app downloaded onto their mobile phones and receive a detailed orientation on its use from a trained research coordinator. Participants will be instructed to input their carbohydrate intake at meals, gestational weight gain, blood glucose levels, and blood pressure into the GDM-DH app, and the GDM-DH app will track the daily number of steps taken by the user. The GDM-DH app will automatically generate feedback through visual charts to help users understand how their health data relate to recommendations. Users can use this information to set goals and self-manage their GDM. The GDM-DH app will also send in-app notifications and reminders to input data and attend prenatal care appointments. During standard care prenatal visits, clinicians will review data from the app via a web portal to inform treatment decisions and recommendations. Approved family members can also be added to the app and can track patient progress.

### Outcome Measures

#### Perinatal Health Outcomes

Exploratory maternal and neonatal health outcomes will be measured at delivery and 6 weeks post partum (Table 1). Primary exploratory treatment outcomes will be maternal glycemic control measures at 6 weeks post partum, neonatal birth weight, and rates of labor induction and cesarean delivery. As secondary exploratory treatment outcomes, we will also look at gestational weight gain, glucose levels above the glycemic targets and rates of insulin therapy, neonatal hypoglycemia, and admittance to the neonatal intensive care unit (NICU).

**Table 1:** Study outcomes and covariates for phase 2, proof-of-concept randomized controlled trial.

Outcome variable				Data source		Collection timepoint						
						Baseline		36-38 weeks gestation		Delivery		6-weeks post partum
**Maternal outcomes**												
	Fasting and 2-hour blood glucose		Medical record		✓						✓	
	Gestational weight gain		Medical record						✓			
	Glucose readings during gestation		GDM-DH^a^ app and medical record (intervention group); medical record (standard care group)						✓			
	Initiation of insulin therapy		Medical record						✓			
	Induction of labor		Medical record						✓			
	Cesarean delivery		Medical record						✓			
**Neonatal outcomes**												
	Birth weight		Medical record						✓			
	Apgar score		Medical record						✓			
	NICU^b^ admission		Medical record						✓			
	Neonatal hypoglycemia		Medical record						✓			
**Usability and acceptability outcomes**												
	Oxford Maternity Diabetes Treatment Satisfaction Questionnaire [56]		Survey (self-report)				✓					
**Variables measured in the intervention group only**												
	**App usage**		GDM-DH app									
		Time spent on the app									✓	
	**Self-monitoring compliance (observed vs expected number of entries)**		GDM-DH app									
		Carbohydrate intake									✓	
		Blood glucose entries										
		Blood pressure entries										
		Weight entries										
	**mHealth^c^ App Usability Questionnaire [57]**		Survey (self-report)				✓					
	**System Usability Scale** **[54]**		Survey (self-report)				✓					
**Covariates**												
	Sociodemographics		Survey (self-report)		✓							
	Prepregnancy weight		Survey (self-report)		✓							

^a^GDM-DH: gestational diabetes mellitus-Dhulikhel Hospital.

^b^NICU: neonatal intensive care unit.

^c^mHealth: mobile health.

At 6 weeks (±5 days) post partum, all participants will undergo a standard 75 g oral glucose tolerance test. Fasting and 2-hour glucose levels will be measured in the hospital laboratory using standard laboratory methods, and we will abstract these measures from the medical record. Blood glucose measures from the routine screening for GDM at 24 to 28 weeks of gestation will also be abstracted from the medical record. Additional maternal outcome variables will be collected at delivery. Induction of labor (yes or no), cesarean delivery (yes or no), and initiation of insulin therapy (yes or no) will be abstracted from the medical records. Gestational weight gain measures will be abstracted from the medical record and calculated by subtracting the measured weight at or before 12 weeks gestation from the measured weight at delivery. Maternal gestational glucose readings will be extracted from the app and medical record (for the intervention group) or the medical record (standard care group), and the proportions and frequency of glucose levels above the glycemic targets (≤5.5 mmol/L preprandial and ≤6.6 mmol/L at 2 hours postprandial) will be calculated. Neonatal outcome measures will also be collected at delivery. Prior to discharge (<24 hours after delivery), neonatal birth weight will be abstracted from the medical record. Neonatal Apgar score, NICU admission (yes or no), and neonatal hypoglycemia (yes or no) will be abstracted from the medical record.

#### Usability and Acceptability

At 36 to 38 weeks gestation, we will use the Oxford Maternity Diabetes Treatment Satisfaction Questionnaire, a validated 9-item 7-point Likert scale questionnaire (+3= strongly agree to –3= strongly disagree; possible scores 0-27) that can be completed in under 5 minutes, to assess general satisfaction and acceptability of GDM care [56]. Good acceptability will be considered predominantly (>80%) neutral or positive scores on all 9 items. The questionnaire will also include space for free text responses, where participants will be encouraged to provide additional feedback or suggestions.

For participants randomized to the study intervention group, we will collect additional measures. Questionnaires, which can be completed in under 5 minutes, will be administered by a trained research assistant at 36 to 38 weeks gestation to assess app usability and acceptability. The SUS will be used to assess perceived app usability, with a score of 68 (out of 100) demonstrating good usability [54]. Usability will also be assessed through a secondary mHealth App Usability Questionnaire (MAUQ) [57]. The MAUQ is a 7-point, 21-item survey designed to assess patient feedback on mHealth apps and has been previously validated. The MAUQ comprises of 3 subscales—ease of use and satisfaction (8 items, questions 1-8); system information arrangement (6 items, questions 9-14); and usefulness (7 items, questions 15-21), with responses to questions ranging on a 7-point scale from 1 (strongly agree) to 7 (strongly disagree). Lower scores indicate superior performance or a more positive user experience compared to higher scores. In addition to app usability and acceptability, we will collect data about app usage and self-monitoring frequency (eg, the actual number of app entries or expected app entries multiplied by 100 for carbohydrate intake or blood glucose). These data will be collected post partum from the GDM-DH app.

### Sample Size Estimation and Considerations

Treatment-related change in glycemic control is of primary interest to this study, and we will be adequately powered to examine whether mean change in fasting and 2-hour blood glucose levels from 24 to 28 weeks gestation (collected at enrollment) to 6 weeks post partum differed significantly by the treatment condition. Power analyses were estimated based on repeated measures ANOVA with in-between interaction using G*Power (version 3.1.9.2; Heinrich Heine University Düsseldorf). For an estimated medium effect size of 0.25 (partial eta squared =0.06), an α error level of .05, a nonsphericity correction of 1, and to test 2 groups with 2 repeated measurements with a correlation of 0.5, a sample size of 34 is required to achieve the power of 80%. We will recruit an additional 86 patients (total n=120) to account for possible attrition and to test other hypotheses with exploratory clinical outcomes. Prior RCTs evaluating mHealth solutions for GDM management detected significant differences in treatment arms with similar or smaller sample sizes [58-60]. In 2017, the number of live births in Dhulikhel Hospital was 2983. Using a conservative incidence rate of 5%, the expected number of newly diagnosed patients with GDM per month is 12. Anticipating a conservative recruitment rate of 50%, we expect it will take 20 months to recruit 120 patients with GDM for the study.

### Statistical Analysis

Data analysis will be done using SAS (version 9.4; SAS Institute). Maternal and infant characteristics will be described overall and by treatment condition using descriptive statistics (mean, SDs for continuous variables or frequencies for categorical variables). Independent 2-tailed *t* tests or chi-square analyses will examine differences in birth weight or rates of labor induction or cesarean delivery, respectively, between the 2 treatment conditions [61,62]. Usability and acceptability measures reported by the study intervention group will be summarized using descriptive statistics. A repeated measures ANOVA will be used to investigate whether fasting and 2-hour blood glucose levels differed across the 2 timepoints (within effect), and more importantly, whether the mean changes in fasting and 2-hour blood glucose levels over time (24 to 28 weeks gestation to 6 weeks post partum) differed by the 2 treatment conditions (interaction effect) [63]. Two separate models will be tested for fasting and 2-hour blood glucose levels, respectively. Assumptions of sphericity will be evaluated using Mauchly’s [64] test statistic for all models. A *P* value of <.05 will be considered statistically significant for all analyses.

## Results

As of July 2024, we have completed phase 1 of the study. Phase 2 is underway. The first participant was enrolled in the RCT in October 2021, with 99 participants enrolled as of July 2024. We anticipate completing recruitment by December 2024 and disseminating the findings by December 2025.

## Discussion

### Principal Findings

This protocol describes the study methodology to develop a user-centered and culturally tailored GDM-DH platform and to determine the usability, acceptability, and preliminary efficacy of the platform among patients with GDM in a university hospital setting in Nepal. The GDM-DH platform will be designed to support patients with GDM in adopting optimal self-management behaviors and assist providers with timely and informed clinical decision-making. We anticipate that the platform will show good usability and acceptability, as well as demonstrate preliminary efficacy for improving perinatal health outcomes, including maternal glycemic control measures at 6 weeks post partum, neonatal birth weight, and rates of labor induction and cesarean delivery. To our knowledge, this will be the first RCT evaluating an intervention that leverages mHealth for GDM treatment and management in an LMIC setting.

Prior research conducted in high-income countries demonstrates that mHealth interventions for GDM, including smartphone apps and web-based tools, promote self-management behaviors, improve glycemic control, and reduce the risks of adverse perinatal outcomes [65-67]. However, existing app-based interventions for GDM management primarily focus on remote glucose monitoring with manual feedback from health care providers [58,59,68,69], which is resource-intensive and burdensome for both providers and participants, thus limiting the potential for widespread dissemination and impact in resource-limited settings. Our platform addresses these challenges by providing automated feedback, which may help to overcome resource limitations and reduce provider burden. While personalized and culturally tailored digital tools are beneficial for enhancing GDM self-management and improving perinatal health [66], few app designs are culturally responsive [70]. The GDM-DH platform improves upon existing mobile interventions for GDM by integrating cultural practices and family influence on prenatal care, offering social support and culturally tailored dietary and physical activity recommendations, and providing automatically generated personalized feedback to optimize GDM management without the need for significant human resources.

### Research and Practice Implications

The GDM-DH app has the potential to significantly improve standard GDM care in Nepal by supporting self-management practices, facilitating informed clinical decision-making, and potentially enhancing clinical outcomes. If our GDM-DH platform proves successful, our findings will be highly relevant to the broader South Asian population and will guide the future development, testing, and optimization of tailored mHealth GDM interventions for other patient groups with low technological sophistication and unique cultural needs. Additionally, the methodologies and lessons learned from designing and implementing the GDM-DH app may inform similar mHealth initiatives in other LMICs.

### Strengths

This study has several strengths and innovative aspects, including the user-centered and culturally tailored design based on behavioral theory, a lifestyle-based self-management approach using technology to minimize user burden, family member involvement, clinician input, and potential to augment prenatal care in a resource-limited setting. The potential for the GDM-DH app to augment prenatal care in resource-limited settings may be particularly significant for countries like Nepal, given the increasing rates of T2D and its comorbidities that consume health care resources [71,72]. Moreover, adequate treatment and management of GDM are critical to disrupting the cycle of intergenerational obesity and diabetes [73], and mHealth may serve as a valuable early-stage intervention strategy to curb the burgeoning T2D epidemic in Nepal and other LMICs.

### Limitations

There are also limitations to the GDM-DH app and study protocol. To minimize user burden, the app collects and provides feedback only on carbohydrate intake, so other aspects of diet (eg, energy intake and fat intake) are not collected. However, the Nepalese diet is characterized by frequent consumption of carbohydrate-rich food sources [74], and carbohydrate intake is a crucial component of GDM self-management [41]. Similarly, the app only records steps taken and does not collect any information about additional aspects of physical activity. Despite this limitation, we expect to capture the majority of physical activity as walking is the most commonly reported exercise during pregnancy [75]. Manual entry of diet, blood glucose, blood pressure, and weight may impact user engagement, as it is time and effort-consuming. However, manual entry may allow participants to be more aware of their behaviors and associated physiological effects, thereby enhancing self-efficacy [40]. Since the app was designed to address the cultural barriers and technological literacy of pregnant women in Nepal, findings may not be generalizable to other women with GDM. If the app is efficacious, a similar process to adapt and culturally tailor the app to other settings and populations may be followed. Finally, this study may not be adequately powered to test secondary clinical outcomes (eg, NICU admission). However, we will be able to explore the trends and treatment differences in secondary outcomes, enabling us to power a larger study.

### Future Directions and Dissemination

In a future study, we will conduct a type 1 or type 2 effectiveness-implementation trial to test the clinical and cost-effectiveness of the GDM-DH platform in improving GDM treatment outcomes. We will make every effort to keep the technologies (GDM-DH platform) developed as a result of this research project, if any, widely available and accessible to the research community.

### Conclusions

With increasing rates of GDM, particularly in resource-limited settings, there is a heightened interest in developing innovative approaches to augment the treatment and management of this common pregnancy complication. App-based lifestyle interventions for GDM management are not common, especially in LMICs where GDM prevalence is rapidly increasing. This protocol describes the study methodology for developing and testing an mHealth platform designed to manage and treat GDM in Nepal. This proof-of-concept trial will garner important information about leveraging mobile technology for GDM management in LMICs and holds important public health relevance for the broader South Asian population.

## Appendix

Multimedia Appendix 1 Peer-review report.

## Abbreviations

GDM: gestational diabetes mellitus
GDM-DH: gestational diabetes mellitus–Dhulikhel Hospital
HIPAA: Health Insurance Portability and Accountability Act
LMIC: low- and middle-income country
MAUQ: Mobile Health App Usability Questionnaire
mHealth: mobile health
MVP: minimum viable product
NICU: neonatal intensive care unit
OB-GYN: obstetrician-gynecologist
RCT: randomized controlled trial
SCT: Social Cognitive Theory
SUS: System Usability Scale
T2D: type 2 diabetes

## Data Availability

The data sets generated and analyzed during this study will be available from the corresponding author upon reasonable request.
