# Supplementary material for: Development and Testing of a Mobile App for Management of Gestational Diabetes in Nepal: Protocol for a User-Centered Design Study and Exploratory Randomized Controlled Trial
Source: JMIR Res Protoc. 2024 Oct 21;13:e59423. doi: 10.2196/59423 (PMC11535795; doi:10.2196/59423)
Supplement: Multimedia Appendix 1 [file resprot_v13i1e59423_app1.pdf]

SUMMARY STATEMENT
(Privileged Communication)

PROGRAM CONTACT:
Laura Povlich
301-496-1653
povlichlk@mail.nih.gov

Release Date: **03/22/2019**
Revised Date:

Application Number: **1 R21 TW011377-01**

Principal Investigator

RAWAL, SHRISTI

Applicant Organization: RBHS-SCHOOL/ HEALTH RELATED PROFESSIONS

Review Group: **ZRG1 HDM-A (55)**
Center for Scientific Review Special Emphasis Panel
PAR-16-292 Mobile Health: Technology and Outcomes in Low and Middle Income Countries

Meeting Date: **03/01/2019**
Council: **MAY 2019**
Requested Start: **07/01/2019**
RFA/PA: **PAR18-242**
PCC: **MHEALTH**

Project Title: **Development and Testing of a Mobile Health Application for Management of Gestational Diabetes**
SRG Action: **Impact Score:30 Percentile:15 #**
Next Steps: **Visit https://grants.nih.gov/grants/next_steps.htm**
Human Subjects: **30-Human subjects involved - Certified, no SRG concerns**
Animal Subjects: **10-No live vertebrate animals involved for competing appl.**
Gender: **2A-Only women, scientifically acceptable**
Minority: **5A-Only foreign subjects, scientifically acceptable**
Children: **3A-No children included, scientifically acceptable**

Project Year	Direct Costs Requested	Estimated Total Cost
1	125,000	192,012
2	100,000	153,610
TOTAL	225,000	345,622

1R21TW011377-01 Rawal, Shristi

RESUME AND SUMMARY OF DISCUSSION: This application proposes to develop and test the usability and preliminary efficacy of a smartphone application-based lifestyle intervention to support self-management of gestational diabetes mellitus (GDM) among patients in Nepal. During discussion, the Editors agreed that the proposal addresses a significant and emerging public health concern in low- and middle-income countries (LMICs) and the goals of the study are supported by a relatively well constructed set of scientific premises. If successful, findings may inform strategies to improve glycemic control and reduce adverse pregnancy outcomes in woman with GDM. The investigative team is outstanding thought there is concern regarding unbalanced expertise at different study sites. Additional strengths of the proposal include a strong conceptual framework, rigorous study design and robust plans to build mHealth research capacity at the partnering LMIC institution. During discussion, however, the editors identified issues that detract somewhat from these strong points. The feasibility of bringing patients back for follow up is not adequately considered and it not clear that the proposed follow-up timeline is sufficient to establish glycemic control. These issues notwithstanding, overall, findings are expected to have a high to moderate impact on prenatal care for woman with gestational diabetes mellitus in resource limited settings.

DESCRIPTION (provided by applicant): The prevalence of gestational diabetes mellitus (GDM) is rapidly increasing worldwide, particularly in low- and middle-income countries (LMICs). Tight glycemic control via diet and lifestyle modification is critical to treating GDM and preventing its adverse health consequences, including increased risk of type 2 diabetes (T2D), in women and their children. However, in many resource-limited countries including Nepal, time for diet/lifestyle counseling often competes with other components of antenatal care. Mobile health (mHealth) technology can be leveraged to promote healthy behaviors, and support self-management and treatment of GDM, but this approach has not been tried previously in any LMICs. Taking a user-centered design approach, here we propose to develop a culturally- appropriate smartphone application (app) to support self-management of GDM (mGDM), and test its usability and preliminary efficacy, among patients in a tertiary level, university hospital of Kathmandu University, Nepal. Based on the Social Cognitive Theory framework for behavior change, this app will assist in self-management of GDM by increasing the patient's knowledge and self-efficacy to adhere to the recommended diet and physical activity regimens. The app will also help clinicians by generating easily digestible visual displays of patient data and behaviors, which can aid in their clinical decision-making and counseling. In the requirements gathering phase, 6 GDM patients will be recruited into a focus group to view paper prototypes and provide feedback on its features and functions. Additional questions will be asked about their perceived barriers, facilitators, and strategies for lifestyle modification. Key informant interviews will also be conducted with 5 clinicians (gynecologists, dietician, physical therapist) and 3 family members, asking them to provide feedback on the mGDM prototype, especially pertaining to the usefulness and format of the graphic summaries of patient data. After revising paper prototypes and developing the first digital prototype, six additional patients with GDM will be recruited for two rounds of usability testing including think-aloud protocol and focus group discussions. Final prototype will be developed following an iterative process of product design and user testing. After developing the app, we will recruit 60 women who are newly diagnosed with GDM, and randomly assign them to one of two treatment conditions: either (A) mGDM app + standard care, or (B) standard care alone, from 28 weeks of gestation to delivery. In this proof-of-concept trial, feasibility outcomes will be app usage, self-monitoring adherence, and app usability and acceptability. Exploratory treatment outcomes will be glycemic control measures at 6 weeks postpartum, neonatal birthweight, and rates of labor induction and caesarean delivery. Findings from the proposed study will empirically inform a future randomized control trial that will be fully powered to test the efficacy and cost-effectiveness of the mGDM app in improving treatment outcomes, using an R01 mechanism. We expect that the research activities and capacity building initiatives proposed in this application will build the institutional skills and create the organizational structure needed to enable future mHealth research initiatives at Kathmandu University, even beyond the scope of this proposed project.

PUBLIC HEALTH RELEVANCE: Adequate control and management of gestational diabetes mellitus (GDM) during pregnancy is critical to mitigate its short- and long-term health consequences in women and their children and may serve as a key strategy to curb the escalating type 2 diabetes epidemic in low- and middle-income countries (LMICs). Taking a user-centered design approach, here we propose to develop a culturally-appropriate smartphone application (app) to support self-management of GDM, and additionally, test its usability and preliminary efficacy, among patients in a sub-urban hospital setting in Nepal. App-based lifestyle interventions for GDM management are not common, especially in LMICs where its prevalence is rapidly increasing, and as such, our study findings will have important public health relevance for a broader population.

CRITIQUE

EDITORS' IMPACT STATEMENTS:

Overall Impact 1: The prevalence of gestational diabetes mellitus (GDM) is rapidly increasing worldwide, particularly in low- and middle-income countries (LMICs). Tight glycemic control via diet and lifestyle modification is critical to treating GDM and preventing its adverse health consequences, including increased risk of type 2 diabetes (T2D). However, in many resource-limited countries including Nepal, time for diet/lifestyle counseling often competes with other components of clinical care. Mobile health (mHealth) technology can be leveraged to promote healthy behaviors and support self-management and treatment of GDM. Taking a user-centered design approach, the investigators propose to develop a culturally- appropriate smartphone application (app) to support self-management of GDM (mGDM), and test its usability and preliminary efficacy, among patients in a tertiary level, university hospital of Kathmandu University, Nepal. There are two phases of this proposal. In the requirements gathering phase, 6 GDM patients will be recruited into a focus group to view paper prototypes and provide feedback on its features and functions. After revising paper prototypes and developing the first digital prototype, six additional patients with GDM will be recruited for two rounds of usability testing including think-aloud protocol and focus group discussions. Final prototype will be developed following an iterative process of product design and user testing. In the second phase, after developing the app, the investigators will recruit 60 women who are newly diagnosed with GDM, and randomly assign them to one of two treatment conditions: either (A) mGDM app + standard care, or (B) standard care alone, from 28 weeks of gestation to delivery. In this proof-of-concept trial, feasibility outcomes will be app usage, self-monitoring adherence, and app usability and acceptability. Exploratory treatment outcomes will be glycemic control measures at 6 weeks postpartum, neonatal birthweight, and rates of labor induction and caesarean delivery. Based on the Social Cognitive Theory framework for behavior change, this app will assist in self-management of GDM by increasing the patient's knowledge and self-efficacy to adhere to the recommended diet and physical activity regimens. The app will also help clinicians by generating easily digestible visual displays of patient data and behaviors, which can aid in their clinical decision-making and counseling. Therefore, the premise is solid and balanced, and the study is poised to resolve a long-standing issue of gestational diabetes mellitus (GDM) in Nepal. There are a few minor weaknesses, including unbalanced experiences in different facilities, and institutional support; and 6 weeks glycemic control may not be good enough. However, the overall impact and significance outweigh the minor weaknesses.

Overall Impact 2: This project seeks to develop and test the usability, acceptability, and preliminary effectiveness of a mobile app for the management of gestational diabetes in Nepal, where there is a growing prevalence of pregnancies complicated by GDM. The project is of high importance given the significant poor outcomes for both babies and their mothers who develop this condition. The advantage of a mobile app for providing a lifestyle intervention in Nepal, given the country's mobile service penetration is over 100%. The investigators, while mostly junior in the states, and more senior in Nepal, seem capable of successfully accomplishing all study aims. The project is innovative, as such mHealth innovations have not been introduced into Nepal, despite the mobile service penetration. Consistent

with the RFA guidelines, the investigators propose an entire aim of creating an organizational framework to strengthen mhealth research capacity at Katmandu University, where some of their project team worked together and successfully launched Nepal's first patient-centered health education app. The project is based on an appropriate theoretical framework, social cognitive theory, which suggests that increased self-efficacy and social support serve as mediators leading to improved self-management of GDM. The analytic plan is strong. Timeline is acceptable and there are no problems related to the clinical trial. Given these strengths, this proposal has a high likelihood of having a major impact, especially when combined with the strong preexisting relationship between investigators at the partnering institutions.

Overall Impact 3: The topic under study is woman with gestational diabetes and an mHealth strategy to help achieve glycemic control through diet and life style modifications to reduce adverse pregnancy outcomes. Gestational diabetes, which can adversely affect the mother and baby, is a growing concern in South Asia. The team has strong complementary expertise to support the PI (who is Nepalese). The behavior change intervention is guided by a strong theoretical framework (social cognitive theory) but not all relevant variables that influence diet appear to be included; feasibility metrics are robust. The collaboration seems to have strong potential for sustainability and capacity building (university and teaching hospital partners in Nepal). Minor concerns have to do with the inclusion of women with diabetes but are otherwise low risk. The sample size may not have sufficient power to detect actual behavior change (the baseline measurement is unclear as well) but will be able to describe feasibility. While there are other mobile health interventions in this context, this proposal has sufficient rigor and premise that findings have high potential for impact. Human subjects: protections adequately described; the exclusion of pregnant females under 18 is not a concern for this feasibility study.

MAIL REVIEWERS' CRITIQUES:

CRITIQUE 4

Significance: 1
Investigator(s): 1
Innovation: 1
Approach: 2
Environment: 1

Overall Impact: This well-conceived proposal that builds upon prior collaborative research with Rutgers University in chronic disease monitoring and management. The mHealth applications to be developed, field tested and utilized will focus on a patient centered approach to woman with gestational diabetes to help achieve tight glycemic control through diet and life style modifications in the hope of reducing adverse pregnancy outcomes.

1. Significance:

Strengths

- South Asia has become the diabetes hub of the world. Interventions that in low-resource settings like Nepal, where specialized antepartum care is limited, must rely heavily on consumer education, and on cost-effective ways of support patient practices to improve pregnancy outcomes.
- The far-reaching aims described in the proposal, if proven feasible and acceptable will provide a nidus for scale up.
- The potential for disrupting the intergenerational cycle of risk for type II diabetes and its longer-term sequelae is compelling.

Weaknesses

- No weaknesses seen.

2. Investigator(s):

Strengths

- The PI is a Nepalese researcher on the facility of Rutgers University, with a background in epidemiology. She has a history of collaboration on research directly linked to diabetes, cardiovascular disease, obesity and the knowledge of local dietary patterns.
- The team that has been assembled is stellar in its background with expertise in mHealth technology, high risk obstetrics, nutritional science, physical activity and risk reduction, obesity research and community health and provides a balance between US and Nepalese investigators.

Weaknesses

- Given the vast and relevant experience of Dr. Pagoto, it is unlikely that 52 hours of consulting time will be sufficient.
- The team may benefit from additional biostatistical expertise.

3. Innovation:

Strengths

- The promotion of optimal health behaviors to gestational diabetic women via mHealth in low/middle income countries has not previously been described.
- The user-centered design approach consistent with Social Cognitive Theory is an innovative cost-effective initiative particularly appropriate for under-resourced settings. The monitoring of app usage, adherence and practical usability/ acceptability will provide critical feasibility findings necessary to a devise larger intervention trial.

Weaknesses

- No weaknesses seen.

4. Approach:

Strengths

- While limited in scope, the “pilot” phase of the proposal seeks to assure appropriate input and adherence to local and cultural norms.
- There is sufficient expertise in app development among us research collaborators. However, partnering with a commercial firm within Nepal will strengthen mHealth capability and increase infrastructure to support future research.

Weaknesses

- The clinical trial outcomes of glycemic control at 6 weeks post-partum, neonatal birthweight and rates of labor induction and caesarean deliveries may not be well correlated with tight glycemic control.
- The relative low number of women in the trial component may not allow for adequate trend data.

5. Environment:

Strengths

- The support of the Nepal Health Research Council of Nepal, Kathmandu University and the central role of its primary teaching hospital supports an appropriate environment to conduct a proof of consent study which will incorporate 60 women, newly diagnosed with gestational diabetes. It will employ a newly developed mHealth app and compare these with standard of care alone to determine clearly stated pre-specific outcomes.

Weaknesses

- It is not clear if the recruitment will occur at outpatient clinics linked to the hospital or among hospitalized patients.
- The percent of women/families that have access to smartphones is not mentioned.

Study Timeline:

Strengths

- The study timeline is appropriate.

Weaknesses

- None noted.

Protections for Human Subjects:

Acceptable Risks and/or Adequate Protections

Data and Safety Monitoring Plan (Applicable for Clinical Trials Only):

Acceptable

Inclusion of Women, Minorities and Children:

- Sex/Gender: Distribution justified scientifically
- Race/Ethnicity: Distribution justified scientifically
- For NIH-Defined Phase III trials, Plans for valid design and analysis: Not applicable
- Inclusion/Exclusion of Children under 18: Excluding ages <18; justified scientifically

Vertebrate Animals:

Not Applicable (No Vertebrate Animals)

Biohazards:

Not Applicable (No Biohazards)

Applications from Foreign Organizations:

Justified

Select Agents:

Acceptable

Resource Sharing Plans:

Acceptable

Authentication of Key Biological and/or Chemical Resources:

Not Applicable (No Relevant Resources)

Budget and Period of Support:

Recommend as Requested

CRITIQUE 5

Significance: 3
Investigator(s): 5
Innovation: 4
Approach: 4
Environment: 4

Overall Impact: This project proposes to develop a culturally appropriate smartphone application (app) to support self-management of gestational diabetes mellitus (GDM), and test its usability and preliminary efficacy, among patients in a tertiary level, university hospital of Kathmandu University, Nepal. Based on the Social Cognitive Theory framework for behavior change, this app will assist in self-management of GDM by increasing the patient's knowledge and self-efficacy to adhere to the recommended diet and physical activity regimens. The premise is solid and balanced and the study is poised to resolve a long-standing issue of gestational diabetes mellitus (GDM) in Nepal. The proposed studies of use of mobile app and theoretical framework do not offer a notable advantage over studies already done, and relevance to the success is questionable. Even if successful, it is unclear that these results would move the field of mobile app. At Nepali site, the facility has the experience of using mobile app. The facilities at Nepal site was not described well and it is unclear whether the university can fully support the high demands associated with this mHealth study.

1. Significance:

Strengths

- The premise is solid and balanced, and the study is poised to resolve a long-standing issue of gestational diabetes mellitus (GDM) in Nepal.
- Preliminary results support the value of using the mobile app to study GDM. These studies will provide specific insights into the role of mobile app in GDM.
- This application seeks to develop a mobile app and advance current knowledge of taking care of GDM. If successful, such understanding of GDM would further allow use of theoretical framework for GDM to be accomplished, which would have a high impact on the field of new born babies and taking care of pregnant woman.

Weaknesses

- The proposed studies of use of the mobile app and theoretical framework do not offer a notable advantage over studies already done, and relevance to the success is questionable. Even if successful, it is unclear that these results would move the field of mobile app.
- The application lacks justification that use of the app over text messaging as use of app always need to be on but text messaging is better controlled by the doctors.

2. Investigator(s):

Strengths

- Over the past couple of years, the PI has published relevant papers in this area but the PI does not see patients like a medical doctor.
- The consultant in theoretical framework is highly accomplished in the field of behavior change.
- Co-investigator in Nepal has firsthand experience.

Weaknesses

- The experience of the PI for conducting these complicated studies is not extensive. He does not appear to have significant expertise to provide guidance for critical aspects such as use of the theoretical framework and mobile app.
- The team appears to lack expertise in mobile app development, particularly important for completing Aims 1 and 2.
- The investigators have not worked together as a team.

3. Innovation:

Strengths

- The combination of mobile app and theoretical foundation is distinct from other approaches in mHealth to study GDM.

Weaknesses

- Although the project uses new methods such as theoretical framework and mobile app, these methods are unlikely to generate different conceptual approaches related to theoretical framework than what currently drives mHealth.
- The concept of use of app is not new to the field.
- The research question is a modest extension of the investigator's existing work and does not move in new directions.
- Continued use of the well-established mobile app will yield only incremental additional knowledge.

4. Approach:

Strengths

- Specific Aim 2 was well-designed to test the main hypothesis. The investigators proposed well-controlled, appropriately powered set of rigorous experiments that addresses the impact of mobile app within this model system.
- The experimental design is comprehensive and cohesively covers all aspects of use of mobile app.

Weaknesses

- The approach to develop the mobile app lacks any methodology like agile software development.
- Use of text messaging along with the mobile app would improve the outcome.

5. Environment:

Strengths

- The environment at Rutgers is outstanding because it has all of the necessary environment to build the app.
- At Nepal site, the facility has the experience of using mobile app.

Weaknesses

- The facilities at Nepal site were not described and it is unclear whether the university can fully support the high demands associated with this mHealth studies.

Study Timeline:

Strengths

- The time line is well justified along with the app development and getting feedback.

Weaknesses

- None.

Protections for Human Subjects:

Acceptable Risks and/or Adequate Protections

Data and Safety Monitoring Plan (Applicable for Clinical Trials Only):

Acceptable

Inclusion of Women, Minorities and Children:

- Sex/Gender: Distribution justified scientifically
- Race/Ethnicity: Distribution justified scientifically
- For NIH-Defined Phase III trials, Plans for valid design and analysis: Not applicable
- Inclusion/Exclusion of Children under 18: Excluding ages <18; justified scientifically

Vertebrate Animals:

Not Applicable (No Vertebrate Animals)

Biohazards:

Not Applicable (No Biohazards)

Applications from Foreign Organizations:

Justified

Select Agents:

Not Applicable (No Select Agents)

Resource Sharing Plans:

Acceptable

Authentication of Key Biological and/or Chemical Resources:

Not Applicable (No Relevant Resources)

Budget and Period of Support:

Recommend as Requested

CRITIQUE 6

Significance: 1
Investigator(s): 2
Innovation: 2
Approach: 1
Environment: 1

Overall Impact: The overall impact of this project is likely to be high based on several strengths and only a few weaknesses. There is a need for this type of MHealth app to overcome limitations in health resources for management of GDM in LMIC – a gap that also exists in HIC but for which there is active research. The team, although comprised of several relatively junior investigators with some key senior collaborators, appears to have the expertise and drive to see the project through to completion. The environment is strong on both sides, and the likelihood of building capacity for future mHealth is particularly strong given a) the senior expertise in Nepal and prior work; b) the specific inclusion of a plan within Aim 3. The scientific aims for development of the smart phone app and testing within a pilot study seems appropriate, although recruitment criteria should also include women with GDM but otherwise low risk pregnancy.

1. Significance:

Strengths

- This study will address a significant and emerging problem in LMIC – GDM.
- The study appropriately identifies the key constraints in LMIC for counseling and care of women during pregnancy (and although they don’t address the primary constraint which is universal screening/testing (!) they are working in a hospital with universal testing). One of the constraints to testing is what to do about it if you find it and this app may provide a feasible/acceptable management strategy.
- They create a cogent argument for this type of app and one can see the generalizability of such an app in other settings in other countries. Thus, if development, found feasible/acceptable and with future testing, this could be an important addition to prenatal care for women with GDM in LMIC.
- Although not mentioned, and absent glucose monitoring the app could be expanded as a primary resource on diet and physical activity during pregnancy for women as part of prenatal care who do not have GDM.
- The partnership and prior relationships between KU and Rutgers (and the investigators) and prior app development in Nepal to be expanded here enhance the significance of this project from the perspective of capacity building in mHealth.

Weaknesses

- There are no identified weaknesses.

2. Investigator(s):

Strengths

- The team has the appropriate expertise to carry out the project, including expertise in GDM/obstetrics/antenatal care in Nepal, diet and lifestyle in Nepal, app based PA monitoring, app development including smartphone based development, SCT/behavior change within the context of mHealth and app; scientific coordination at high levels with Nepal, research grant administration, etc.

Weaknesses

- Several members of the research team are quite junior, the PI is in first year of faculty position at Rutgers. This is not necessarily a concern, as the career trajectories of the junior investigators is quite impressive, and it is balanced by senior collaborators, particularly at KU.

3. Innovation:

Strengths

- The investigators appropriately argue that their planned development of an app which emphasizes dietary and physical activity monitoring as management of GDM in an environment with barriers to glucose monitoring and high clinical follow up.
- The app will provide an innovative approach to capture physical activity which has not been tested previously in LMIC.
- The extent to which they will develop content for an app for women, who may be vegetarian and whose carbohydrate intake may be close to 70%, is new, needed and of potential generalizability throughout South Asia.

Weaknesses

- It may be argued that the project is not innovative because it may be more about moving prenatal GDM monitoring from paper and pamphlets (SOC, which we don’t know the content or quality of – although it might be good) to a mobile app.

4. Approach:

Strengths

- The plan/set of activities for strengthening mHealth research in Nepal is strong, plausible and has high potential for continued growth beyond the project period.
- The approach for the app development are appropriate in sequence and accompanying research methodologies (Focus groups/key informant interviews, think out loud methodology and pilot testing compared to SOC).
- Their approach will build on prior results (which we do not much about them) regarding the diet and physical activity of women in Nepal. They will address guiding women in knowledge and self-efficacy regarding carbohydrate sources and assessment.
- They recognize the need to consider caste.
- These last 2 points add to the significance and likely overall impact of the project in that there is experience in the rather quick adaption of diet related materials/app in Nepal to the context of India.

Weaknesses

- The goals and sample size calculation for the pilot focusing on change in glucose challenge (prenatal to postpartum) is a stretch, as if the researchers are trying for a health outcome. Having said that the sample size and approach of the pilot to examine usability of the app and collection of data on biologic variables is appropriate, and the pilot results should inform the design of a larger trial to test the app.

5. Environment:

Strengths

- The environment appears appropriate for the project. They have identified the landscape for mHealth in Nepal, and although not everyone has one, they are likely to be on the rise.
- There is shared experience in the development of smartphone app for health through another project, and prior collaborations amongst the group.
- There is appropriate resources and structure in Nepal to carry out this project, in terms of scientific and medical resources, and technical expertise in app development.

Weaknesses

- There are no recognized weaknesses

Study Timeline:

Strengths

- The timeline seems appropriate for app development, which is largely related to content development and user considerations, and the execution of the pilot trial is justified with data/evidence by the applicant.
- The PI has an ongoing study with some of the other investigators on diet and physical activity of women in Nepal and thus likely has local content which can be adapted for this project. They are not starting from scratch.
- The strength of the environment and the prior collaborations allow for this timeline.

Weaknesses

- The timeline of app development (9 months at most) and recruitment for the pilot (6 months) may be optimistic.

Protections for Human Subjects:

Acceptable Risks and/or Adequate Protections

- This is a minimal risk study of a smartphone app as an alternate (better) support for women with GDM within the context of ongoing prenatal care.

Data and Safety Monitoring Plan (Applicable for Clinical Trials Only):

Acceptable

- The description seems appropriate given the level of risk and the ongoing care of the participants in the pilot (Aim 2).

Inclusion of Women, Minorities and Children:

- Sex/Gender: Distribution justified scientifically
- Race/Ethnicity: Distribution justified scientifically
- For NIH-Defined Phase III trials, Plans for valid design and analysis: Not applicable
- Inclusion/Exclusion of Children under 18: Excluding ages <18; not justified scientifically
- They will not recruit women with GDM during pregnancy who are less than 18, which I take to be age of majority in Nepal. There is no justification for this, although it could be argued that a pregnant woman < 18 with GDM would be so unusual as to detract from the scientific purpose of obtaining pilot data.

Vertebrate Animals:

Not Applicable (No Vertebrate Animals)

Biohazards:

Not Applicable (No Biohazards)

Applications from Foreign Organizations:

Not Applicable (No Foreign Organizations)

Select Agents:

Not Applicable (No Select Agents)

Resource Sharing Plans:

Not Applicable (No Relevant Resources)

Authentication of Key Biological and/or Chemical Resources:

Not Applicable (No Relevant Resources)

Budget and Period of Support:

Recommend as Requested

THE FOLLOWING SECTIONS WERE PREPARED BY THE SCIENTIFIC REVIEW OFFICER TO SUMMARIZE THE OUTCOME OF DISCUSSIONS OF THE REVIEW COMMITTEE, OR REVIEWERS' WRITTEN CRITIQUES, ON THE FOLLOWING ISSUES:

PROTECTION OF HUMAN SUBJECTS: ACCEPTABLE

INCLUSION OF WOMEN PLAN: ACCEPTABLE

INCLUSION OF MINORITIES PLAN: ACCEPTABLE

INCLUSION OF CHILDREN PLAN: ACCEPTABLE

COMMITTEE BUDGET RECOMMENDATIONS: The budget was recommended as requested.

Footnotes for 1 R21 TW011377-01; PI Name: Rawal, Shristi

Ad hoc or special section application percentiled against "Total CSR" base.

NIH has modified its policy regarding the receipt of resubmissions (amended applications). See Guide Notice NOT-OD-14-074 at http://grants.nih.gov/grants/guide/notice-files/NOT-OD-14-074.html. The impact/priority score is calculated after discussion of an application by averaging the overall scores (1-9) given by all voting reviewers on the committee and

multiplying by 10. The criterion scores are submitted prior to the meeting by the individual reviewers assigned to an application, and are not discussed specifically at the review meeting or calculated into the overall impact score. Some applications also receive a percentile ranking. For details on the review process, see http://grants.nih.gov/grants/peer_review_process.htm#scoring.

MEETING ROSTER

Center for Scientific Review Special Emphasis Panel
CENTER FOR SCIENTIFIC REVIEW
PAR-16-292 Mobile Health: Technology and Outcomes in Low and Middle Income Countries

ZRG1 HDM-A (55)
03/01/2019

Notice of NIH Policy to All Applicants: Meeting rosters are provided for information purposes only. Applicant investigators and institutional officials must not communicate directly with study section members about an application before or after the review. Failure to observe this policy will create a serious breach of integrity in the peer review process, and may lead to actions outlined in NOT-OD-14-073 at https://grants.nih.gov/grants/guide/notice-files/NOT-OD-14-073.html and NOT-OD-15-106 at https://grants.nih.gov/grants/guide/notice-files/NOT-OD-15-106.html, including removal of the application from immediate review.

CHAIRPERSON(S)

KLESGES, ROBERT C, PHD
PROFESSOR
DEPARTMENT OF PUBLIC HEALTH SCIENCES
CENTER FOR ADDICTION PREVENTION RESEARCH
SCHOOL OF MEDICINE
UNIVERSITY OF VIRGINIA
CHARLOTTESVILLE, VA 22911

MEMBERS

BARCLAY, GILLIAN R, DDS, DPH, MPH
VICE PRESIDENT, PROGRAMS
WALDEN UNIVERSITY
MINNEAPOLIS, MN 55401

DE ERAUSQUIN, GABRIEL ALEJANDRO, MD, PHD
PROFESSOR AND FOUNDING CHAIR
DEPARTMENT OF PSYCHIATRY AND NEUROLOGY
SCHOOL OF MEDICINE
UNIVERSITY OF TEXAS RIO GRANDE VALLEY
BROWNSVILLE, TX 78520

DOEBBELING, BRADLEY NIEL, MD
PROFESSOR
SCIENCE OF HEALTH CARE DELIVERY
AND BIOMEDICAL INFORMATICS
COLLEGE OF HEALTH SOLUTIONS
ASU DOWNTOWN PHOENIX CAMPUS
PHOENIX, AZ 85004

DYKES, PATRICIA C, PHD
ASSOCIATE PROFESSOR
PROGRAM DIRECTOR CENTER FOR NURSING EXCELLENCE
CENTER FOR PATIENT SAFETY RESEARCH AND PRACTICE
BRIGHAM AND WOMEN'S HOSPITAL
HARVARD MEDICAL SCHOOL
BOSTON, MA 02120

EHRHARDT, STEPHAN, MD, MPH
ASSOCIATE PROFESSOR
DEPARTMENT OF EPIDEMIOLOGY
JOHNS HOPKINS SCHOOL OF PUBLIC HEALTH
BALTIMORE, MD 21205

ESWARAN, HARI, PHD
PROFESSOR
DEPARTMENT OF OBSTETRICS AND GYNECOLOGY
UNIVERSITY OF ARKANSAS FOR MEDICAL SCIENCES
LITTLE ROCK, AR 72205

IYENGAR, MADURAI SRIRAM, PHD
ASSOCIATE PROFESSOR OF BIOMEDICAL INFORMATICS
COLLEGE OF MEDICINE
TEXAS A AND M HEALTH SCIENCE CENTER
HOUSTON, TX 77030

LEVKOFF, SUE E, SCD, SM
PROFESSOR AND ENDOWED CHAIR
SENIORSMART CENTER OF ECONOMIC EXCELLENCE
COLLEGE OF SOCIAL WORK
UNIVERSITY OF SOUTH CAROLINA
COLUMBIA, SC 29201

LI, LANG, PHD
PROFESSOR AND CHAIR
DEPARTMENT OF BIOMEDICAL INFORMATICS
COLLEGE OF MEDICINE
THE OHIO STATE UNIVERSITY
COLUMBUS, OH 43210

MENON, USHA, PHD
PROFESSOR AND VICE DEAN OF RESEARCH
COLLEGE OF NURSING
UNIVERSITY OF SOUTH FLORIDA
TAMPA, FL 33612-4766

ODAME, KOFI, PHD
ASSOCIATE PROFESSOR
ENGINEERING SCIENCES
THAYER SCHOOL OF ENGINEERING
DARTMOUTH COLLEGE
HANOVER, NH 03755

PHILIS-TSIMIKAS, ATHENA, MD
CORPORATE VICE PRESIDENT
ENDOCRINOLOGY, DIABETES & METABOLISM
SCRIPPS WHITTIER DIABETES INSTITUTE
LA JOLLA, CA 92037

SOLIMAN, AMR, MD, PHD
PROFESSOR
DEPARTMENT OF COMMUNITY HEALTH AND SOCIAL MEDICINE
CUNY SCHOOL OF MEDICINE
NEW YORK, NY 10031

WARRICK, CYNTHIA A, PHD
PRESIDENT
STILLMAN COLLEGE
TUSCALOOSA, AL 35401

WOLINSKY, FREDRIC D, SCD, PHD
PROFESSOR AND COLLOTON CHAIR, EMERITUS
DEPARTMENT OF HEALTH MANAGEMENT AND POLICY
COLLEGE OF PUBLIC HEALTH
THE UNIVERSITY OF IOWA
IOWA CITY, IA 52242

XIAN, XIAOJUN, PHD
RESEARCH SCIENTIST
CENTER FOR BIOELECTRONICS AND BIOSENSORS
THE BIODESIGN INSTITUTE
ARIZONA STATE UNIVERSITY
TEMPE, AZ 85287

MAIL REVIEWER(S)

ADAMSON, LAUREN B, PHD
REGENTS' PROFESSOR EMERITA
DEPARTMENT OF PSYCHOLOGY
COLLEGE OF ARTS AND SCIENCES
GEORGIA STATE UNIVERSITY
ATLANTA, GA 30303

AHAMED, SHEIKH IQBAL, PHD
PROFESSOR
DEPARTMENT OF MATHEMATICS, STATISTICS AND COMPUTER SCIENCE
MARQUETTE UNIVERSITY
MILWAUKEE, WI 53233

ANDERSON, PAGE L, PHD
ASSISTANT PROFESSOR
DEPARTMENT OF PSYCHOLOGY
GEORGIA STATE UNIVERSITY
ATLANTA, GA 30303

ANWAR, MOHD, PHD
ASSOCIATE PROFESSOR
THE SECURE USABLE SOCIAL MEDIA AND NETWORKS LAB
NORTH CAROLINA A AND T STATE UNIVERSITY
GREENSBORO, NC 27411

ARCOLEO, KIMBERLY JOAN, PHD
ASSOCIATE PROFESSOR
ASSOCIATE DEAN FOR RESEARCH
SCHOOL OF NURSING
UNIVERSITY OF ROCHESTER
ROCHESTER, NY 14642

AREAN, PATRICIA A, PHD
PROFESSOR
DEPARTMENT OF PSYCHIATRY AND BEHAVIORAL SCIENCES
SCHOOL OF MEDICINE
UNIVERSITY OF WASHINGTON
SEATTLE, WA 98102

ARONSON, IAN DAVID, PHD
PRINCIPAL INVESTIGATOR
CENTER FOR TECHNOLOGY AND BEHAVIORAL HEALTH
NATIONAL DEVELOPMENT AND RESEARCH INSTITUTES, INC
NEW YORK, NY 10010

ASCHE, CARL, MSC, PHD
PROFESSOR
COLLEGE OF MEDICINE
UNIVERSITY OF ILLINOIS
PEORIA, IL 61615

BARRIO, CONCEPCION, PHD
ASSOCIATE PROFESSOR
SCHOOL OF SOCIAL WORK
UNIVERSITY OF SOUTHERN CALIFORNIA
LOS ANGELES, CA 90089

BAUM, MARIANNA K, PHD
PROFESSOR
DEPARTMENT OF DIETETICS AND NUTRITION
ROBERT STEMPEL COLLEGE OF PUBLIC HEALTH AND SOCIAL WORK
FLORIDA INTERNATIONAL UNIVERSITY
MIAMI, FL 33199

BECK, JOHN ROBERT, MD
DEPUTY DIRECTOR
DEPARTMENT OF CANCER PREVENTION AND CONTROL
FOX CHASE CANCER CENTER
PHILADELPHIA, PA 19111

BOIVIN, MICHAEL JOSEPH, MPH, PHD
PROFESSOR
DEPARTMENTS OF PSYCHIATRY AND NEUROLOGY/OPHTHALMOLOGY
COLLEGE OF OSTEOPATHIC MEDICINE
MICHIGAN STATE UNIVERSITY
EAST LANSING, MI 48824

BONATO, PAOLO, PHD
ASSOCIATE PROFESSOR
SPAUDLING REHABILITATION HOSPITAL
MOTION ANALYSIS LABORATORY
HARVARD MEDICAL SCHOOL
BOSTON, MA 02114

BOWMAN, NATALIE MCCARTER, MPH, MD
ASSISTANT PROFESSOR OF MEDICINE
SCHOOL OF MEDICINE
UNIVERSITY OF NORTH CAROLINA CHAPEL HILL
CHAPEL HILL, NC 27516

BREITBART, WILLIAM, MD
PROFESSOR AND CHIEF OF PSYCHIATRY SERVICE
DEPARTMENT OF PSYCHIATRY
AND BEHAVIORAL SCIENCES
SLOAN-KETTERING INSTITUTE FOR
CANCER RESEARCH
NEW YORK, NY 10022

CALLAWAY, CLIFTON W, MD, PHD
PROFESSOR AND EXECUTIVE VICE-CHAIR
DEPARTMENT OF EMERGENCY MEDICINE
UNIVERSITY OF PITTSBURGH
PITTSBURGH, PA 15261

CAMPBELL, FRANCES A, PHD
SENIOR SCIENTIST
FRANK PORTER GRAHAM CHILD DEVELOPMENT CENTER
UNIVERSITY OF NORTH CAROLINA
CHAPEL HILL, NC 27599-8180

CAULFIELD, LAURA E, PHD
PROFESSOR
CENTER FOR HUMAN NUTRITION
DEPARTMENT OF INTERNATIONAL HEALTH
THE JOHNS HOPKINS BLOOMBERG
SCHOOL OF PUBLIC HEALTH
BALTIMORE, MD 21205

CHEN, YING QING, PHD
FULL MEMBER
PROGRAM IN BIOSTATISTICS
VACCINE AND INFECTIOUS DISEASE DIVISION
FRED HUTCHINSON CANCER RESEARCH CENTER
SEATTLE, WA 98109

COOK, DIANE JOYCE, PHD
HUIE-ROGERS CHAIR PROFESSOR
SCHOOL OF ELECTRICAL ENGINEERING
AND COMPUTER SCIENCE
WASHINGTON STATE UNIVERSITY
PULLMAN, WA 99164

DAGOGO-JACK, SAMUEL, MBBS, MD
A C MULLINS CHAIR IN TRANSLATIONAL RESEARCH
PROFESSOR OF MEDICINE AND DIRECTOR
DIVISION OF ENDOCRINOLOGY, DIABETES AND
METABOLISM
THE UNIVERSITY OF TENNESSEE HEALTH SCIENCE
CENTER
MEMPHIS, TN 38163

DATAR, ASHLESHA, PHD
SENIOR ECONOMIST
DIRECTOR OF PROGRAM ON CHILDREN AND FAMILIES
CENTER FOR ECONOMIC AND SOCIAL RESEARCH (CESR)
UNIVERSITY OF SOUTHERN CALIFORNIA
LOS ANGELES, CA 90089

DERMAN, RICHARD J, MD, MPH
ASSOCIATE PROVOST, GLOBAL AFFAIRS
DIRECTOR, GLOBAL HEALTH RESEARCH
PROFESSOR, OBSTETRICS AND GYNECOLOGY
THOMAS JEFFERSON UNIVERSITY
PHILADELPHIA, PA 19017

DIAMANTIDIS, CLARISSA JONAS, MD
ASSISTANT PROFESSOR
SCHOOL OF MEDICINE
DUKE UNIVERSITY
DURHAM, NC 27701

DUERR, ANN C, MD, PHD
AFFILIATE PROFESSOR
HIV VACCINE TRIALS NETWORK
FRED HUTCHINSON CANCER RESEARCH CENTER
DEPARTMENTS OF EPIDEMIOLOGY AND GLOBAL HEALTH
UNIVERSITY OF WASHINGTON
SEATTLE, WA 98109

EDDY, JOHN MARK, PHD
DIRECTOR OF COMMUNITY BASED RESEARCH
AND SENIOR RESEARCH SCIENTIST
FAMILY TRANSLATIONAL RESEARCH GROUP
DEPARTMENT OF CARDIOLOGY AND COMPREHENSIVE
CARE
NEW YORK UNIVERSITY COLLEGE OF DENTISTRY
NEW YORK, NY 10010

ERIKSEN, MICHAEL PAUL, SCD
PROFESSOR AND DIRECTOR
INSTITUTE OF PUBLIC HEALTH
GEORGIA STATE UNIVERSITY
ATLANTA, GA 30302

FARMER, ELIZABETH M, PHD
PROFESSOR AND DEAN OF THE SCHOOL OF SOCIAL WORK
UNIVERSITY OF PITTSBURGH
UNIVERSITY PARK, PA 16802

FOX, WILLIAM CHRISTOPHER, MD
ASSOCIATE PROFESSOR; PROGRAM DIRECTOR
ENDOVASCULAR NEUROSURGERY FELLOWSHIP
ASSOCIATE PROGRAM DIRECTOR, NEUROSURGERY
RESIDENCY
DEPARTMENT OF NEUROSURGERY
UNIVERSITY OF FLORIDA
GAINESVILLE, FL 32610

FRENCH, DUSTIN D, PHD
SENIOR RESEARCH SCIENTIST
DEPARTMENT OF OPTHALMOLOGY, NORTHWESTERN
UNIVERSITY FEINBERG SCHOOL OF MEDICINE
SENIOR RESEACH SCIENTIST
EDWARD HINES, JR VA HOSPITAL
CHICAGO, IL 60141

FULLER, SHERRILYNNE S, PHD
PROFESSOR EMERITUS
DEPARTMENT OF BIOMEDICAL INFORMATICS
AND MEDICAL EDUCATION
SCHOOL OF MEDICINE AND INFORMATION SCHOOL
UNIVERSITY OF WASHINGTON
SEATTLE, WA 98195

GONG, YANG, PHD, MD
ASSOCIATE PROFESSOR
SCHOOL OF BIOMEDICAL INFORMATICS
UNIVERSITY OF TEXAS HEALTH SCIENCE
CENTER AT HOUSTON
HOUSTON, TX 77030

HALL, DREW ALEXANDER, PHD
ELECTRICAL AND COMPUTER ENGINEERING
UNIVERSITY OF CALIFORNIA, SAN DIEGO
LA JOLLA, CA, CA 92093

HANSEN, MATTHEW LEE, MD
ASSOCIATE PROFESSOR
DEPARTMENT OF EMERGENCY MEDICINE
OREGON HEALTH AND SCIENCE UNIVERSITY
PORTLAND, OR 97239

HERSH, WILLIAM R, MD
PROFESSOR AND CHAIR
DEPARTMENT OF MEDICAL INFORMATICS
AND CLINICAL EPIDEMIOLOGY
SCHOOL OF MEDICINE
OREGON HEALTH AND SCIENCE UNIVERSITY
PORTLAND, OR 97239

INAN, OMER TOLGA, PHD
ASSISTANT PROFESSOR
SCHOOL OF ELECTRICAL AND COMPUTER ENGINEERING
GEORGIA INSTITUTE OF TECHNOLOGY
ATLANTA, GA 30308

IRAZUZTA, JOSE E, MD
PROFESSOR
DIRECTOR OF EDUCATION AND RESEARCH
DEPARTMENT OF PEDIATRICS
DIVISION OF PEDIATRIC CRITICAL CARE
UNIVERSITY OF FLORIDA
JACKSONVILLE, FL 32207

JO, JAVIER ANTONIO, MS, PHD
PROFESSOR OF ELECTRICAL AND COMPUTER
ENGINEERING
STEPHENSON RESEARCH AND TECHNOLOGY CENTER
THE UNIVERSITY OF OKLAHOMA
NORMAN, OK 73072

JOHNSON, HEATHER M, MD
ASSOCIATE PROFESSOR
DIVISION OF CARDIOVASCULAR MEDICINE
DEPARTMENT OF MEDICINE
UNIVERSITY OF WISCONSIN SCHOOL OF MEDICINE
AND PUBLIC HEALTH
MADISON, WI 53792

KOHLER, HANS-PETER, PHD
PROFESSOR OF DEMOGRAPHY AND ECONOMICS
DEPARTMENT OF SOCIOLOGY
UNIVERSITY OF PENNSYLVANIA
PHILADELPHIA, PA 19104

KOHRT, BRANDON ALAN, MD, PHD
ASSOCIATE PROFESSOR
PSYCHIATRY AND BEHAVIORAL SCIENCES
GEORGE WASHINGTON UNIVERSITY
SCHOOL OF MEDICINE AND HEALTH SCIENCES
WASHINGTON, DC 20037

KUSHNIRUK, ANDRE WILLIAM, PHD
DIRECTOR AND PROFESSOR
SCHOOL OF HEALTH INFORMATION SCIENCE
UNIVERSITY OF VICTORIA
VICTORIA, BRITISH COLUMBIA V8W 2Y2
CANADA

LIU, LONGJIAN, MD, PHD
ASSOCIATE PROFESSOR
DEPARTMENT OF EPIDEMIOLOGY AND BIOSTATISTICS
DREXEL UNIVERSITY
PHILADELPHIA, PA 19102

MA, GRACE X, PHD
ASSOCIATE DEAN FOR HEALTH DISPARITIES
DIRECTOR, CENTER FOR ASIAN HEALTH, LAURA H CARNEL
PROFESSOR AND PROFESSOR IN CLINICAL SCIENCE
LEWIS KATZ SCHOOL OF MEDICINE
TEMPLE UNIVERSITY
PHILADELPHIA, PA 19122

MANNE, SHARON L, MA, PHD
ASSOCIATE DIRECTOR, CANCER PREVENTION, CONTROL,
AND POPULATION SCIENCE
RUTGERS CANCER INSTITUTE OF NEW JERSEY
ROBERT WOOD JOHNSON MEDICAL SCHOOL
UNIVERSITY OF MEDICINE AND DENTISTRY NEW JERSEY
NEW BRUNSWICK, NJ 08903

MOHAN, SUMIT, MPH, MBBS
ASSOCIATE PROFESSOR OF EPIDEMIOLOGY AND MEDICINE
MAILMAN SCHOOL OF PUBLIC HEALTH
COLUMBIA UNIVERSITY MEDICAL CENTER
NEW YORK , NY 10032

MURPHY, ROBERT L, MD
PROFESSOR OF MEDICINE AND BIOMEDICAL ENGINEERING
CENTER FOR GLOBAL HEALTH
NORTHWESTERN UNIVERSITY
CHICAGO, IL 60611

NACHEGA, JEAN BISIMWA, MD, PHD
ASSOCIATE PROFESSOR
DEPARTMENTS OF EPIDEMIOLOGY, INFECTIOUS DISEASES,
AND MICROBIOLOGY
SCHOOL OF PUBLIC HEALTH
UNIVERSITY OF PITTSBURGH
PITTSBURGH, PA 15261

OGUNSEITAN, OLADELE A, MPH, PHD
PROFESSOR
DEPARTMENT OF PUBLIC HEALTH AND SOCIAL ECOLOGY
COLLEGE OF HEALTH SCIENCES
SCHOOL OF SOCIAL ECOLOGY
UNIVERSITY OF CALIFORNIA, IRVINE
IRVINE, CA 92697

PASUPATHY, KALYAN S, PHD
ASSOCIATE PROFESSOR
MAYO COLLEGE OF MEDICINE
SCIENTIFIC DIRECTOR
CLINICAL ENGINEERING LEARNING LAB
MAYO CLINIC
ROCHESTER, MN 55905

POELLABAUER, CHRISTIAN, PHD
ASSOCIATE PROFESSOR
DEPARTMENT OF COMPUTER SCIENCE
AND ENGINEERING
UNIVERSITY OF NOTRE DAME
NOTRE DAME, IN 46556

POSTMUS, JUDY L, PHD
PROFESSOR AND DIRECTOR
SCHOOL OF SOCIAL WORK, RUTGERS UNIVERSITY
CENTER ON VIOLENCE AGAINST WOMEN AND CHILDREN
DUNELLEN, NJ 08812

QIN, LIDONG, PHD
PROFESSOR
DEPARTMENT OF NANOMEDICINE
DEPARTMENT OF CELL AND DEVELOPMENTAL BIOLOGY
HOUSTON METHODIST RESEARCH INSTITUTE
HOUSTON, TX 77030

QUINTILIANI, LISA M, PHD
ASSISTANT PROFESSOR
DEPARTMENT OF MEDICINE
SECTION OF GENERAL INTERNAL MEDICINE
BOSTON UNIVERSITY
BOSTON, MA 02118

RANGACHARI, PAVANI, PHD
ASSOCIATE PROFESSOR
DEPARTMENT OF HEALTH MANAGEMENT AND INFORMATICS
ASSOCIATE DEAN FOR PRACTICE AND COMMUNITY HEALTH
COLLEGE OF ALLIED HEALTH SCIENCES
GEORGIA HEALTH SCIENCES UNIVERSITY
AUGUSTA, GA 30912

SCHMELER, KATHLEEN, MD
ASSOCIATE PROFESSOR
DEPARTMENT OF GYNECOLOGIC ONCOLOGY AND REPRODUCTIVE MEDICINE
UT MD ANDERSON CANCER CENTER
HOUSTON, TX 77230

SHAW, RYAN JEFFREY, MS, PHD
ASSOCIATE PROFESSOR
SCHOOL OF NURSING
CENTER FOR HEALTH INFORMATICS
CENTER FOR PRECISION MEDICINE
DUKE UNIVERSITY
DURHAM, NC 27708

SHERMAN, SCOTT E, MD, MPH
PROFESSOR
DEPARTMENT OF POPULATION HEALTH, MEDICINE AND PSYCHIATRY
VA NEW YORK HARBOR HEALTHCARE SYSTEM
NEW YORK UNIVERSITY SCHOOL OF MEDICINE
NEW YORK, NY 10016

SLUSHER, TINA MARYE, MD
PROFESSOR OF PEDIATRICS CRITICAL CARE
DIVISION OF GLOBAL PEDIATRICS PARK AVENUE G7
UNIVERSITY OF MINNESOTA
MINNESOTA, MN 55415

STAIANO, AMANDA E, PHD
ASSISTANT PROFESSOR
PENNINGTON BIOMEDICAL RESEARCH CENTER
LOUSIANA STATE UNVERSITY
BATON ROUGE, LA 70808

STAVRINOS, DESPINA, PHD
ASSISTANT PROFESSOR
DEPARTMENT OF PSYCHOLOGY
UNIVERSITY OF ALABAMA AT BIRMINGHAM
BIRMINGHAM, AL 35294

STEEL, JENNIFER L, PHD
ASSOCIATE PROFESSOR
DIRECTOR, CENTER FOR EXCELLENCE IN BEHAVIORAL MEDICINE
DEPARTMENT OF SURGERY
UNIVERSITY OF PITTSBURGH
PITTSBURGH, PA 15213

SUBICA, ANDREW MAKOTO, PHD
ASSISTANT PROFESSOR IN RESIDENCE
UNIVERSITY OF CALIFORNIA, RIVERSIDE
SOM CENTER, HEALTHY COMMUNITIES
RIVERSIDE, CA 92521

SWARD, KATHERINE ANN, RN, PHD
ASSOCIATE PROFESSOR
DEPARTMENT OF BIOMEDICAL INFORMATICS
COLLEGE OF NURSING
UNIVERSITY OF UTAH
SALT LAKE CITY, UT 84112

THOMAS, JAMES CONLEY, PHD
ASSOCIATE PROFESSOR OF PEDIATRICS AND MOLECULAR BIOLOGY
UNIVERSITY OF SOUTH CAROLINA
CHAPEL HILL, NC 27599

TURNER, ANNE M, MD
ASSOCIATE PROFESSOR
SCHOOL OF PUBLIC HEALTH
HEALTH SERVICES
BIOMEDICAL INFORMATICS AND MEDICAL EDUCATION
UNIVERSITY OF WASHINGTON
SEATTLE, WA 98105

WADE, ERIC, PHD
ASSISTANT PROFESSOR
DEPARTMENT OF MECHANICAL, AEROSPACE, AND BIOMEDICAL ENGINEERING
UNIVERSITY OF TENNESSEE, KNOXVILLE
KNOXVILLE, TN 37996

WALANI, SALIMAH R, MPH, PHD
DIRECTOR
GLOBAL HEALTH PROGRAMS
MARCH OF DIMES
WHITE PLAINS, NY 10605

WAN, XIUFENG HENRY, PHD
PROFESSOR
DEPARTMENT OF BASIC SCIENCES
COLLEGE OF VETERINARY MEDICINE
MISSISSIPPI STATE UNIVERSITY
MISSISSIPPI STATE, MS 39762

WETHINGTON, ELAINE, PHD
PROFESSOR
DEPARTMENT OF HUMAN DEVELOPMENT
BRONFENBRENNER CENTER FOR TRANSLATIONAL RESEARCH
COLLEGE OF HUMAN ECOLOGY
CORNELL UNIVERSITY
ITHACA, NY 14853

WILDER-SMITH, PETRA E, LDS RCS E, DDS, PHD
PROFESSOR
DIRECTOR OF DENTAL PROGRAM
BECKMAN LASER INSTITUTE
SCHOOL OF MEDICINE
UNIVERSITY OF CALIFORNIA, IRVINE
IRVINE, CA 92612

SCIENTIFIC REVIEW OFFICER

VOSVICK, MARK ALLEN, PHD
SCIENTIFIC REVIEW OFFICER
CENTER FOR SCIENTIFIC REVIEW
NATIONAL INSITUTES OF HEALTH
BETHESDA, MD 20892

EXTRAMURAL SUPPORT ASSISTANT

BUTLER, SEAN
LEAD - EXTRAMURAL SUPPORT ASSISTANT
CENTER FOR SCIENTIFIC REVIEW
NATIONAL INSTITUTE OF HEALTH
BETHESDA, MD 20892

OTHER REVIEW STAFF

TINKER, REBECCA I, PHD
SCIENTIFIC REVIEW STAFF (CONTRACTOR)
CENTER FOR SCIENTIFIC REVIEW
NATIONAL INSTITUTES OF HEALTH
BETHESDA, MD 20817

Consultants are required to absent themselves from the room during the review of any application if their presence would constitute or appear to constitute a conflict of interest.
